# Assessment of differences in immune responses to albumin compared with crystalloids for resuscitation in sepsis

**DOI:** 10.1186/s13054-026-05954-6

**Published:** 2026-06-18

**Authors:** John Cafferkey, Jennifer Rynne, Andrew Ferguson, Julia Grahamslaw, Peter Smith, Ewan A. M. Pow, Katherine Oatey, Daniel Horner, Alasdair R. Corfield, Alasdair Gray, Manu Shankar-Hari

**Affiliations:** 1 Emergency Medicine Research Group (EMERGE), Royal Infirmary of Edinburgh, 51 Little France Crescent, Edinburgh, EH16 4SA UK; 2 Centre for Inflammation Research, Institute of Regeneration and Repair, University of Edinburgh, Edinburgh, UK; 3 Biomedical Teaching Organisation (BMTO), Edinburgh Medical School, College of Medicine and Veterinary Medicine, University of Edinburgh, Edinburgh, UK; 4 Edinburgh Clinical Trials Unit, Usher Institute, University of Edinburgh, Edinburgh, UK; 5 Emergency Department, Northern Care Alliance NHS Foundation Trust, Salford, UK; 6 Division of Infection, Immunity and Respiratory Medicine, The University of Manchester, Manchester, UK; 7 Emergency Department, Royal Alexandra Hospital, Paisley, UK; 8 University of Glasgow, Glasgow, UK; 9 Intensive Care Unit, Royal Infirmary of Edinburgh, Edinburgh, UK

## Dear editor,

In sepsis, crystalloid is recommended as first-line resuscitation fluid, however 5% Human Albumin Solution (5% HAS) is also used. 5% HAS purportedly confers immunological benefits including free radical and reactive oxygen species scavenging, plus beneficial effects on neutrophils, cellular anergy and cell death pathways [1]. Our recent feasibility randomised controlled trial reported no statistical difference with use of 5% HAS versus balanced crystalloid (odds ratio 1.50, 95% confidence intervals 0.84 to 2.83 for the 30-day mortality) for the first six hours of resuscitation in patients with sepsis presenting to Emergency Departments (ED) [2].

We sampled a subset of these participants to explore immunological mechanisms. Our aim was to describe differences between the immune cell subsets, cytokines and whole blood transcriptome between treatment groups.

At three participating EDs, patients enrolled into the main trial were approached for enrolment and consented either personally or via personal representative. Blood samples were collected at three timepoints: T1 occurred as soon as possible after enrolment (baseline); T2 at 12 h (± 4 h); and T3 at 24 h (± 6 h) from enrolment. We quantified leucocyte populations via flow cytometry (*BD Biosciences*); performed a custom cytokine panel (*LEGENDplex™*); and conducted whole blood pan-leucocyte transcriptome sequencing (rRNA-depleted, strand-specific RNA-seq; *NEBNext Ultra* II Directional, sequenced on *Illumina* NextSeq 2000 P3). *RStudio* (R version 4.3.1) and packages (including QuSAGE for module analysis) were used for analyses. Significance was denoted by an adjusted p-value (p_adj_) of less than 0.05 and, where relevant, log_2_ fold change of greater than 1.5. Prespecified objectives were exploratory.

26 participants were enrolled with 14 allocated to 5% HAS and 12 to crystalloid. Baseline characteristics were similar between groups and comparable to the main trial [2] by age (mean 68.6 vs. 74.5 years), sex (50% female vs. 58% female), presenting lactate (mean 2.2mmol/L vs. 2.6 mmol/L), initial NEWS score (median 7 vs. 8). By 24 h, the 5% HAS group had received 29.4mL/kg and the crystalloid group 55.4 ml/kg of respective study fluid. Outcomes were similar across groups, occurring less frequently than in the main trial, including critical care admission (7.1% versus 8.3% of participants) and 90-day mortality (7.1% versus 0% of participants).

**Fig. 1:**
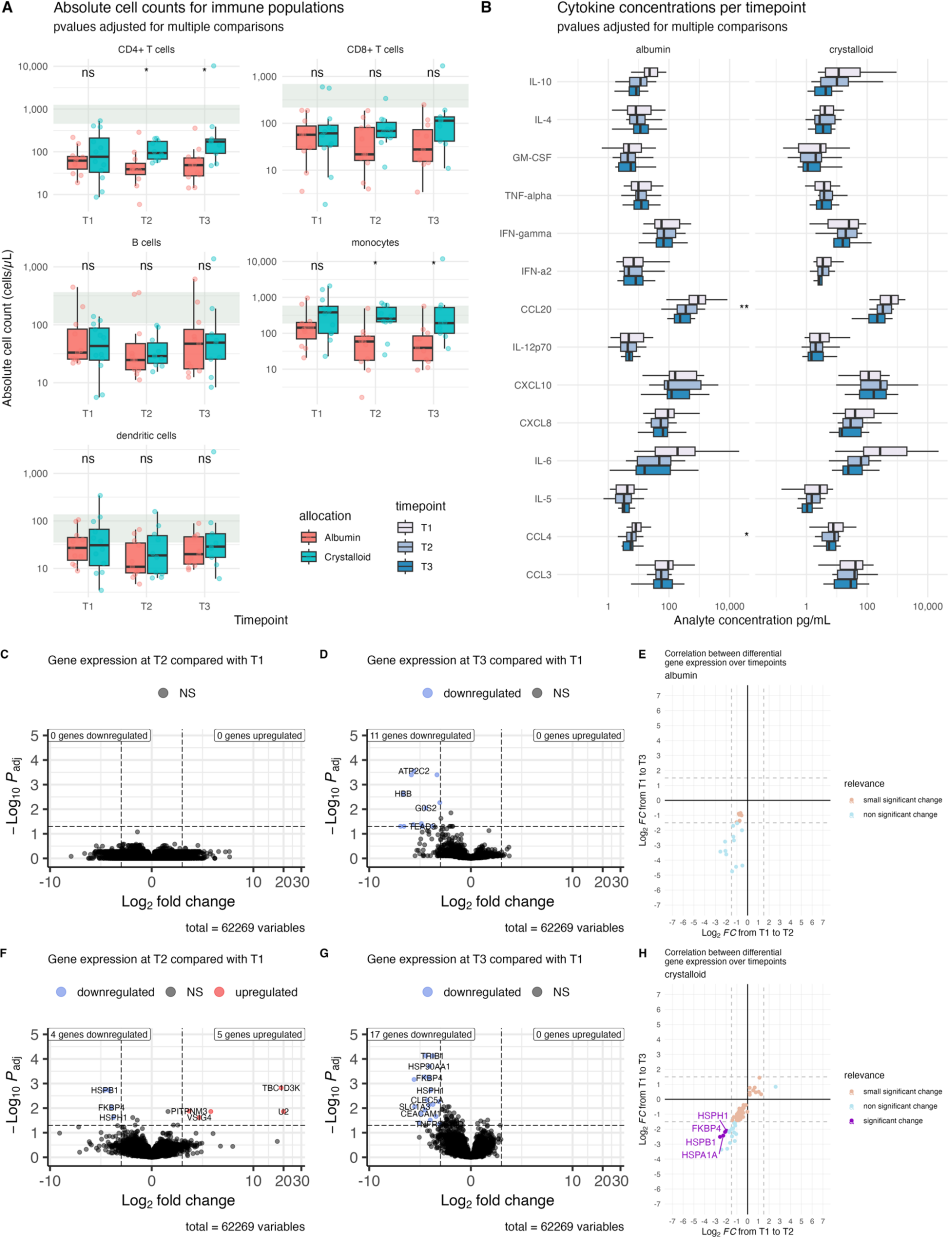
A: Absolute cell counts for relevant populations. ns: Not significant, *:p_adj_ <0.05B: Cytokine concentrations per timepoint. *: p_adj_ <0.05, **: p_adj_ <0.01C, D: Volcano plots of differential expression of genes within the 5% HAS group. E: Quadrant plot for 5% HAS group. Genes that demonstrated statistical significance at any comparison for that group are plotted according to their Log_2_FC (T2 versus T1 on x-axis and T3 versus T1 on y-axis). F, G: Volcano plots of differential expression of genes within the crystalloid group. H: Quadrant plot for crystalloid group, constructed in the same manner as panel "E". Labels are given to genese which met both Log_2_FC and p_adj_ thresholds in both timepoint comparisons for crystalloid. For our data, this occurred exclusively in the lower left quadrant, i.e. where they decreased in T2 and T3 compared with T1.

For immune cell subsets (Fig. 1), monocytes were higher in the crystalloid compared with the 5% HAS group at both T2 (crystalloid 258 vs. 5% HAS 58.9 cells/µL, p_adj_ 0.040) and T3 (192 vs. 39.3, p_adj_ 0.049). A similar effect was seen with CD4 + T-cells increased in the crystalloid group at T2 (94.0 vs. 39.1 cells/µL, p_adj_ 0.040) as well as T3 (173 vs. 48.7 cells/µL, p_adj_ 0.010). No statistically significant trends over time were identified in either group.

For measured cytokines the only significant different point comparison came at T3, where the crystalloid group had significantly lower IFN-gamma compared with 5% HAS (Log_2_FC -2.073, p_adj_ 0.040). However, within the 5% HAS group there were significant decreases over time in CCL20 (T1 to T2 -1480 pg/mL, p_adj_ 0.014; T1 to T3 -1360 pg/mL, p_adj_ 0.009) and CCL4 (T1 to T2 -4.82 pg/mL, p_ad_j 0.028; T1 to T3 -4.49 pg/mL, p_adj_ 0.018). No statistically significant trends occurred in the crystalloid group.

Module analysis of differentially expressed genes (DEG) identified those related to plasma cells, B cells, and immunoglobulins (M156) as expressed significantly less following treatment with crystalloid compared with 5% HAS, comparing between groups at timepoints after treatment. Only within the crystalloid group were there consistently DEG at both T2 and T3 compared with T1, which included heat shock protein coding genes (HSPA1A, HSPB1, and HSPH1) and FKBP4.

Our data generate some interesting observations. 5% HAS was associated with lower CD4 + T-cells and monocytes subsets, which are associated with poorer prognosis via increased risk of secondary infections and death. IFN-gamma, associated with systemic inflammation in sepsis, was lower in the crystalloid group despite higher CD4 + T-cells (the cytokine’s primary source).

Both CCL4 and CCL20 demonstrated within-group reductions for 5% HAS. CCL4 is partially responsible for inducing chemotaxis of T cells, monocytes and natural killer cells, and is associated with survival [3]. CCL20, implicated in chemotaxis of lymphocytes and dendritic cells, is associated with worsening sepsis severity [4], and here low levels of may be explained by the relatively reduced populations of monocytes.

The reduction of heat shock proteins coding genes over time in the crystalloid arm is interesting as they are inducers of both innate and adaptive immunity, and increased circulating heat shock protein levels correlate with increased mortality within sepsis [5].

In summary, we present a uniquely granular window into early sepsis biology. Data are well-balanced, and representative of the main trial and real-life. Small sample size, low incidence of outcomes of interest, and an intervention window of only 6 h are key limitations. Overall, we did not find evidence of significant anti-inflammatory effects with 5% HAS as first-line resuscitation fluid.

## Supplementary Information

Supplementary Material 1

## Data Availability

The datasets used and/or analysed during the current study are available from the corresponding author on reasonable request.

## Abbreviations

5% HAS: 5% Human albumin solution
CCL20: C-C motif chemokine ligand 20
CCL3: C-C motif chemokine ligand 3
CCL4: C-C motif chemokine 4
CXCL10: C-X-C motif chemokine ligand 10
CXCL8: C-X-C motif chemokine ligand 8
DEG: Differentially expressed genes
ED: Emergency Department
GM-CSF: Granulocyte-macrophage colony stimulating factor
IFN-a2: Interferon alpha 2
IFN-gamma: Interferon gamma
IL-10: Interleukin 10
IL-12p70: Interleukin 12p70
IL-4: Interleukin 4
IL-5: Interleukin 5
IL-6: Interleukin 6
Log_2_FC: Log_2_ fold change
T1: Timepoint 1 (enrolment)
T2: Timepoint 2 (12 ± 4 h)
T3: Timepoint 3 (24 ± 6 h)
TNF-alpha: Tumour necrosis factor alpha
