# Supplementary material for: Assessment of differences in immune responses to albumin compared with crystalloids for resuscitation in sepsis
Source: Crit Care. 2026 Jun 18;30:315. doi: 10.1186/s13054-026-05954-6 (PMC13281611; doi:10.1186/s13054-026-05954-6)
Supplement: Supplementary file 1 — Supplementary Material 1 [file 13054_2026_5954_MOESM1_ESM.docx]

MIS ABC SEPSIS SUPPLEMENTARY MATERIALS

[Recruitment 3](#_Toc223526131)

[Main study PICO structure 3](#_Toc223526132)

[Main study inclusion and exclusion criteria 3](#_Toc223526133)

[MIS-ABC Sepsis inclusion criteria 4](#_Toc223526134)

[Recruitment arrangements 4](#_Toc223526135)

[Immunophenotyping 6](#_Toc223526136)

[Sample processing 6](#_Toc223526137)

[Data processing and gating 6](#_Toc223526138)

[Cytokine analysis 9](#_Toc223526139)

[Sample processing 9](#_Toc223526140)

[Data processing 9](#_Toc223526141)

[Transcriptome sequencing and analysis 10](#_Toc223526142)

[RNA extraction and QC 10](#_Toc223526143)

[Library preparation 10](#_Toc223526144)

[Library QC 11](#_Toc223526145)

[Sequencing 11](#_Toc223526146)

[Sequencing results 12](#_Toc223526147)

[Transcript-level expression 12](#_Toc223526148)

[Differential expression analysis 12](#_Toc223526149)

[Transcriptomic module analysis 12](#_Toc223526150)

[Analysis within RStudio 14](#_Toc223526151)

[Strobe checklist 16](#_Toc223526152)

[Additional figures 19](#_Toc223526153)

[S1: Data completeness 19](#_Toc223526154)

[S2: Differential expression analysis 20](#_Toc223526155)

[S3: Blood tests 22](#_Toc223526156)

# Recruitment

## Main study PICO structure

The ABC Sepsis PICO structure is described as follows:

- Population: Adult patients with sepsis and a NEWS2 score ≥ 5 requiring IV fluids within one hour of randomisation
- Intervention: IV fluid resuscitation with 5% Human Albumin Solution for the first 6 hours following randomisation
- Comparator: IV fluid resuscitation with balanced crystalloid
- Outcome: Feasibility outcomes of recruitment rate and 30 day mortality

## Main study inclusion and exclusion criteria

The ABC Sepsis inclusion criteria were as follows:

*Adult patients (18 years or older) who present to UK NHS hospitals with community acquired sepsis meeting all of the 5 criteria:*

1. *Clinically suspected or proven infection resulting in principal reason for acute illness;*
2. *NEWS/NEWS2 score ≥5;*
3. *Hospital presentation within last 12hrs;*
4. *Clinician decision has been made that immediate (within 1 hour of assessment) intravenous fluid resuscitation is needed; and;*
5. *Ability to obtain informed consent.*

Exclusion criteria were:

1. *>1 litre of intravenous crystalloid fluid or any intravenous HAS administered prior to eligibility assessment;*
2. *Clinically judged to require immediate surgery (within one hour of eligibility assessment);*
3. *Chronic renal replacement therapy;*
4. *Known allergy/adverse reaction to HAS;*
5. *Known adverse reaction to blood products;*
6. *Palliation/end of life care (explicit decision by patient/family/carers in conjunction with clinical team that any active treatment beyond symptomatic relief is not appropriate);*
7. *Religious beliefs precluding HAS administration;*
8. *Previous recruitment in the trial;*
9. *Known recent severe traumatic brain injury (within 3 months);*
10. *Patients with permanent incapacity;*
11. *Known to have participated in interventional phase of another CTIMP study within the last 30 days.*

## MIS-ABC Sepsis inclusion criteria

A pragmatic approach was used for inclusion criteria into this MIS-ABC Sepsis study. Criteria included:

1. *Able to obtain informed consent*
2. *Eligible for and randomised into the ABC Sepsis trial*

The sole exclusion criterion was:

1. *Excluded from the ABC Sepsis trial*

## Recruitment arrangements

Participants enrolled into the main ABC Sepsis trial were subsequently approached for suitability, informed consent and enrolment into MIS-ABC Sepsis. MIS-ABC Sepsis recruitment was limited by availability of staff for the sampling procedures: participants were enrolled only in sites where MIS-ABC Sepsis infrastructure was set up, at times when the research team would be able to facilitate all sampling time points. MIS-ABC Sepsis recruited for 7 months of the 12 months ABC Sepsis was open and 3 of the 15 sites ABC Sepsis recruited from..

# Immunophenotyping

## Sample processing

Cell immunophenotyping workflows were based on the protocols of Rynne and Fish developed for the REMAP-CAP study^[[1]](#footnote-1)^. Samples were stored as 600µL quantities containing a 1:1 mixture of whole blood and Cytodelics cell stabiliser. Frozen samples were thawed in a water bath, then fixed with Cytodelics solutions which came as part of the cell stabiliser pack. They underwent two lysing steps, again using Cytodelics solutions prepared as per manufacturer’s instruction. They were washed, stained with antibodies from our panel comprising of CD3, CD4, CD8, CD14, CD15, CD16, CD19, CD38, CD58 and HLA-DR, then washed again. Precision Count beads were added immediately before use of the BD 5L Fortessa flow cytometer, along with compensation controls and BD Quantibrite beads used to calculate antigens per cell values.

## Data processing and gating

FCS files were imported into RStudio and metadata relevant to the samples were attached. Data were compensated using compensation controls prior to developing the gating strategy. Quantibrite bead samples were gated initially using FSC.A versus SSC.A scatter plots. Singlets were identified on a FSC.A versus SSC.A plot, and countbeads then identified using one appropriate fluorescent channel versus FSC.A versus SSC.A. Within the singlets population, a cells population was gated on FSC.A versus SSC.A axes. Gating strategy followed the hierarchical gating strategy described by Maecker et al^[[2]](#footnote-2)^. Data were only transformed at the point of plotting populations to decide gates using the “logicle” transformation^[[3]](#footnote-3)^. For HLA-DR, BD Quantibrite samples allowed for calculation of MFI by generation of a standard curve after gating for each of known low, medium low, medium high and high populations, then plotting observed fluorescence of HLA-DR onto the standard curve. Countbeads were used to calculate absolute cell counts as per manufacturers instructions^[[4]](#footnote-4)^. <https://www.biolegend.com/protocols/precision-count-beads-protocol-and-applications/4248/>

The gating hierarchy is summarised in the diagram below:


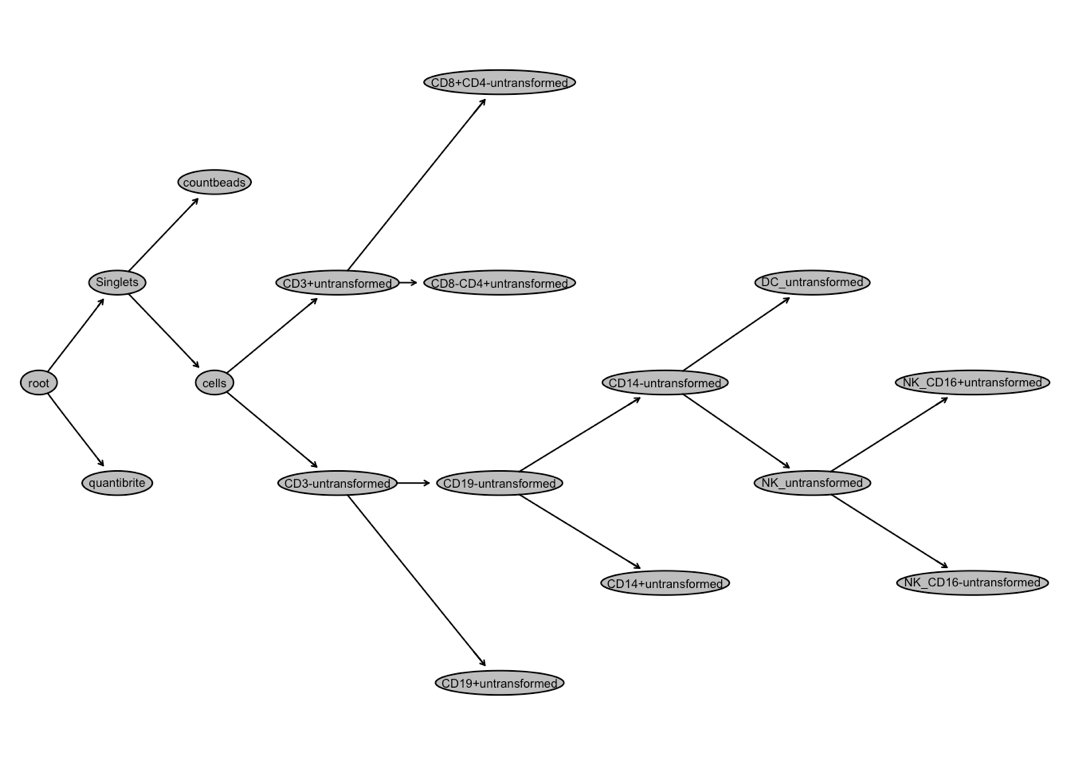


The gating strategy is illustrated below:


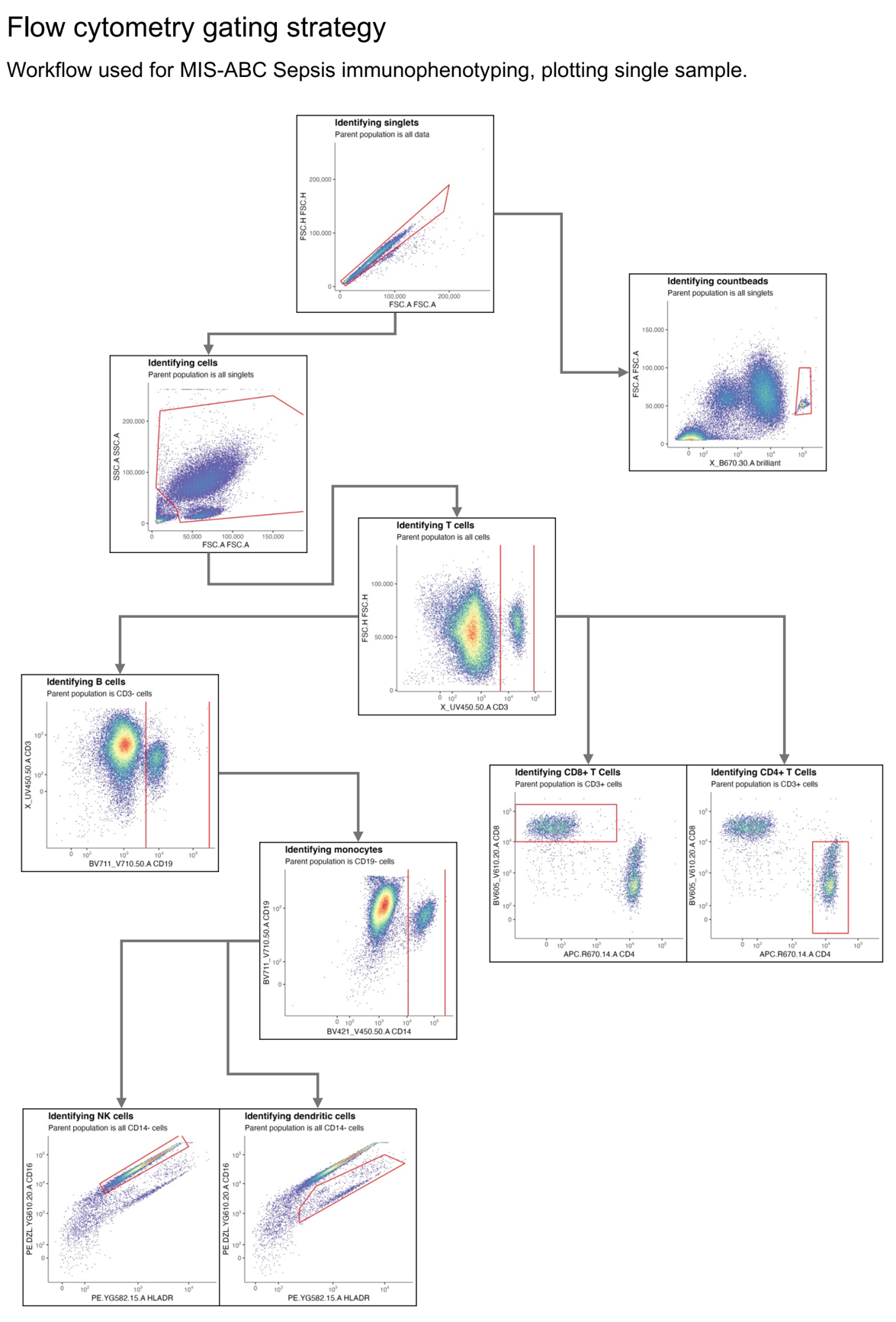


# Cytokine analysis

Cytokine analysis was based upon the COVID-19 Immune Monitoring protocol written by Iva Zlatareva and Biolegend’s LegendPlex assay protocols for the use of the Anti-Viral Response panel (Cat No: 740390) and Human Th panels (Cat No: 740722). Our custom panel comprised of: tumour necrosis factor alpha (TNF-alpha), interleukin 6 (IL-6), granulocyte-macrophage colony-stimulating factor (GM-CSF), C-X-C motif chemokine ligand 10 (CXCL10), interleukin 5 (IL-5), interferon alpha 2 (IFN-a2), interleukin 4 (IL-4), C-C motif chemokine ligand 3 (CCL3), interleukin 10 (IL-10), interleukin 12p70 (IL-12p70), C-C motif chemokine ligand 20 (CCL20), interferon gamma (IFN-gamma), C-X-C motif chemokine ligand 8 (CXCL8), and C-C motif chemokine 4(CCL4).

## Sample processing

Blood was drawn into an EDTA tube, and after aliquoting two 300µL whole blood samples for immunophenotyping, the EDTA tubes were centrifuged at room temperature at 1500G for 10 minutes. From this, up to four aliquots of 300µL plasma were taken and stored at -80ºC until processing. Samples were defrosted until thawed completely, at which point they were diluted two-fold with the appropriate assay buffer. Standards were prepared with serial dilution as per manufacturer’s instructions. Samples were prepared with serial washes as per manufacturer's instructions. The Attune NxT flow cytometer was used after routine set up and quality control procedures. Resulting files were uploaded to the LEGENDplex™ Cloud-based Data Analysis Software hosted by Qognit, with concentrations used for analysis (including those outside the theoretical limit of detection).

## Data processing

Cytokine data were analysed as concentrations in pg/mL in the RStudio environment, with annotated study details and metadata.

# Transcriptome sequencing and analysis

## RNA extraction and QC

Seventy six whole blood samples were provided in either Tempus™ Blood RNA Tubes (Thermo Fisher Scientific, #4342792). Total RNA was extracted from Tempus™ tubes using the MagMAX™ for Stabilised Blood Tubes RNA Isolation Kit (Thermo Fisher Scientific, #4451894). Total RNA samples were assessed on the Fragment Analyser Automated Capillary Electrophoresis System (Agilent Technologies Inc, #5300) with the Standard Sensitivity RNA Analysis Kit (#DNF-471-0500) for quality and integrity of total RNA. ProSize software used to analyse data from the Fragment Analyser system calculates a RNA Quality Number (RQN) for each RNA sample on a scale of 1 to 10 where 1 represents completely degraded total RNA and 10 represents intact total RNA. The majority of RNA samples showed significant signs of degradation (average RQN 3.9). Total RNA was quantified using the Qubit 2.0 Fluorometer (Thermo Fisher Scientific Inc, #Q32866) and the Qubit RNA broad range assay (# Q10210). DNA contamination was quantified using the Qubit dsDNA HS assay kit (#Q32854).

RNA samples with high DNA contamination (>20%) were excluded from the experiment. 69 samples were taken forward to library preparation.

## Library preparation

Libraries were prepared from 250ng of 69 total-RNA samples using the NEBNEXT Ultra II Directional RNA Library Prep kit (New England Biolabs #7760) with NEBNext Globin & rRNA Depletion Kit Human-Mouse-Rat (NEB, #E7550X) modules according to a protocol provided by the manufacturer. Libraries were barcoded using NEBNext Multiplex Oligos for Illumina Unique Dual Index Primer Pairs (NEB E6442L) to increase sample identification specificity and enable complete filtering of index-hopped reads.

Following purification Globin & rRNA-depleted RNA was fragmented using divalent cations under elevated temperature and primed with random hexamers. Primed RNA fragments were reverse transcribed into first strand cDNA using reverse transcriptase and random primers. RNA templates were removed and a replacement strand synthesised incorporating dUTP in place of dTTP to generate ds cDNA. The incorporation of dUTP in second strand synthesis quenches the second strand during amplification as the polymerase used in the assay is not incorporated past this nucleotide. AMPure XP beads (Beckman Coulter, #A63881) were then used to separate the ds cDNA from the second strand reaction mix, providing blunt-ended cDNA. A single 'A' nucleotide was added to the 3' ends of the blunt fragments to prevent them from ligating to another during the subsequent adapter ligation reaction, and a corresponding single 'T' nucleotide on the 3' end of the adapter provided a complementary overhang for ligating the adapter to the fragment. Multiple indexing adapters were then ligated to the ends of the ds cDNA fragments to prepare them for hybridisation onto a flow cell, before 11 cycles of PCR were used to selectively enrich fragments with adapter molecules on both ends and add unique dual index sequences. Post-amplification sequencing libraries were purified using AMPure XP beads.

## Library QC

Libraries were quantified by fluorometry using the Qubit dsDNA HS assay and assessed for quality and fragment size using the Agilent Bioanalyser with the DNA HS Kit (#5067-4626). Fragment size and quantity measurements were used to calculate molarity for each library pool.

## Sequencing

Sequencing was performed using the NextSeq 2000 P3 (200 cycle) Reagents kit (Illumina Inc, #) using P3 flow cells on the NextSeq 2000 platform. Libraries were combined in equimolar pools of 23 based on Qubit and Bioanalyser assay results. PhiX Control v3 Library (Illumina Inc, # FC-110-3001) was spiked in at a concentration of 1% to each run to enable troubleshooting in the event of any instrument-related issue.

## Sequencing results

Three runs were required for this data. Run 3 had sub-optimal data output and quality traced to an issue with lane 2 of the flow cell. Replacement reagents were supplied by Illumina and the run was repeated. There was some variability in the number of clusters PF per library across the runs but all libraries generated ≥41M read pairs if run 3 is omitted (Min: 41.7M, Max: 68.9M, Mean: 53.6M). If run 3 data is included all libraries generated >55M read pairs (Min: 55.7M, Max: 88.0M, Mean: 67.4M).

## Transcript-level expression

FASTQ files were concatenated and then transferred to the Cancer Genomics Cloud (Seven Bridges). The Salmon workflow was used to estimate transcript level abundance, and MultiQC used for QC.

## Differential expression analysis

Transcriptome annotations were loaded using package GenomicFeatures from the gencode.v43 annotation gtf file, and imported with the package tximport. The package DESeq2 was used for differential expression analysis, and gene annotations for graphing were provided from the Ensembl Homo sapiens downloaded using the package biomaRt. Visualisations were created with the help of the EnhancedVolcano and ComplexHeatmap packages.

## Transcriptomic module analysis

Normalised count data on a transcript level were mapped to genes using org.Hs.eg.db Bioconductor package. The issue of multiple transcript IDs relating to a single gene was circumvented by selecting the highest read count for any given gene (i.e. and discarding the read counts for the non-selected transcripts). A counts matrix was constructed and transformed with log_2_ after adding 1 to prevent non-real values. The Blood Transcription Modules^[[5]](#footnote-5)^ was selected and applied to combined data from T2 and T3 (excluding baseline data).

# Analysis within RStudio

Data for all aspects of the analysis were loaded into the RStudio environment (R Core Team (2023). *R: A Language and Environment for Statistical Computing*. R Foundation for Statistical Computing, Vienna, Austria. <https://www.R-project.org/>.). Missing data were not imputed. The following packages were used for the processing, exploration, analysis and visualisation for this project:

| **Package** | **Reference** |
| --- | --- |
| *apeglm* | Zhu, A., Ibrahim, J.G., Love, M.I. Heavy-tailed prior distributions for sequence count data: removing the noise and preserving large  differences Bioinformatics (2018) |
| *BiocManager* | Morgan M, Ramos M (2025). BiocManager: Access the Bioconductor Project Package Repository. R package version 1.30.26, [https://CRAN.R-project.org/package=BiocManager](https://cran.r-project.org/package=BiocManager). |
| *biomaRt* | Mapping identifiers for the integration of genomic datasets with the R/Bioconductor package biomaRt. Steffen Durinck, Paul T.  Spellman, Ewan Birney and Wolfgang Huber, Nature Protocols 4, 1184-1191 (2009). |
| *cluster* | Maechler, M., Rousseeuw, P., Struyf, A., Hubert, M., Hornik, K.(2025). cluster: Cluster Analysis Basics and Extensions. R package  version 2.1.8.1. |
| *ComplexHeatmap* | Gu, Z. (2016) Complex heatmaps reveal patterns and correlations in multidimensional genomic data. Bioinformatics. |
| *cowplot* | Wilke C (2024). cowplot: Streamlined Plot Theme and Plot Annotations for 'ggplot2'. R package version 1.1.3, [https://CRAN.R-project.org/package=cowplot](https://cran.r-project.org/package=cowplot). |
| *DESeq2* | Love, M.I., Huber, W., Anders, S. Moderated estimation of fold change and dispersion for RNA-seq data with DESeq2 Genome Biology  15(12):550 (2014) |
| *edgeR* | Robinson MD, McCarthy DJ and Smyth GK (2010). edgeR: a Bioconductor package for differential expression analysis of digital gene  expression data. Bioinformatics 26, 139-140 |
| *EnhancedVolcano* | Blighe K, Rana S, Lewis M (2023). EnhancedVolcano: Publication-ready volcano plots with enhanced colouring and labeling. doi:10.18129/B9.bioc.EnhancedVolcano <https://doi.org/10.18129/B9.bioc.EnhancedVolcano>, R package version 1.18.0, <https://bioconductor.org/packages/EnhancedVolcano>. |
| *expss* | Demin G (2023). expss: Tables, Labels and Some Useful Functions from Spreadsheets and 'SPSS' Statistics. R package version 0.11.6, [https://CRAN.R-project.org/package=expss](https://cran.r-project.org/package=expss). |
| *factoextra* | Kassambara A, Mundt F (2020). factoextra: Extract and Visualize the Results of Multivariate Data Analyses. R package version 1.0.7, [https://CRAN.R-project.org/package=factoextra](https://cran.r-project.org/package=factoextra). |
| *GenomicFeatures* | Lawrence M, Huber W, Pagès H, Aboyoun P, Carlson M, et al. (2013) Software for Computing and Annotating Genomic Ranges. PLoS Comput  Biol 9(8): e1003118. doi:10.1371/journal.pcbi.1003118 |
| *ggpubr* | Kassambara A (2023). ggpubr: 'ggplot2' Based Publication Ready Plots. R package version 0.6.0, [https://CRAN.R-project.org/package=ggpubr](https://cran.r-project.org/package=ggpubr). |
| *ggrepel* | Slowikowski K (2024). ggrepel: Automatically Position Non-Overlapping Text Labels with 'ggplot2'. R package version 0.9.6, [https://CRAN.R-project.org/package=ggrepel](https://cran.r-project.org/package=ggrepel). |
| *Glimma* | Su, S., Law, C. W., Ah-Cann, C., Asselin-Labat, M. L., Blewitt, M. E., & Ritchie, M. E. (2017). Glimma: interactive graphics for gene  expression analysis. Bioinformatics, 33(13), 2050-2052. |
| *gplots* | Warnes G, Bolker B, Bonebakker L, Gentleman R, Huber W, Liaw A, Lumley T, Maechler M, Magnusson A, Moeller S, Schwartz M, Venables B, Galili T (2024). gplots: Various R Programming Tools for Plotting Data. R package version 3.2.0, [https://CRAN.R-project.org/package=gplots](https://cran.r-project.org/package=gplots). |
| *gridExtra* | Auguie B (2017). gridExtra: Miscellaneous Functions for "Grid" Graphics. R package version 2.3, [https://CRAN.R-project.org/package=gridExtra](https://cran.r-project.org/package=gridExtra). |
| *gtsummary* | Sjoberg DD, Whiting K, Curry M, Lavery JA, Larmarange J. Reproducible summary tables with the gtsummary package. The R Journal  2021;13:570–80. <https://doi.org/10.32614/RJ-2021-053>. |
| *hablar* | Sjoberg D (2023). hablar: Non-Astonishing Results in R. R package version 0.3.2, [https://CRAN.R-project.org/package=hablar](https://cran.r-project.org/package=hablar). |
| *here* | Müller K (2020). here: A Simpler Way to Find Your Files. R package version 1.0.1, [https://CRAN.R-project.org/package=here](https://cran.r-project.org/package=here). |
| *Hmisc* | Harrell Jr F (2025). Hmisc: Harrell Miscellaneous. R package version 5.2-3, [https://CRAN.R-project.org/package=Hmisc](https://cran.r-project.org/package=Hmisc). |
| *limma* | Ritchie, M.E., Phipson, B., Wu, D., Hu, Y., Law, C.W., Shi, W., and Smyth, G.K. (2015). limma powers differential expression analyses for RNA-sequencing and microarray studies. Nucleic Acids Research 43(7), e47. |
| *multcomp* | Hothorn T, Bretz F, Westfall P (2008). “Simultaneous Inference in General Parametric Models.” Biometrical Journal, 50(3), 346-363. |
| *NMF* | Renaud Gaujoux, Cathal Seoighe (2010). A flexible R package for nonnegative matrix factorization. BMC Bioinformatics 2010, 11:367.  [https://bmcbioinformatics.biomedcentral.com/articles/10.1186/1471-2105-11-367] |
| *pathfindR* | Ulgen E, Ozisik O, Sezerman OU. 2019. pathfindR: An R Package for Comprehensive Identification of Enriched Pathways in Omics Data Through Active Subnetworks. Front. Genet. https://doi.org/10.3389/fgene.2019.00858 |
| *QuSAGE* | Yaari G, Bolen C, Thakar J, Kleinstein S (2013). “Quantitative set analysis for gene expression: a method to quantify gene set differential expression including gene-gene correlations.” Nucleic Acids Research 41(18):e170 |
| *RColorBrewer* | Neuwirth E (2022). RColorBrewer: ColorBrewer Palettes. R package version 1.1-3, [https://CRAN.R-project.org/package=RColorBrewer](https://cran.r-project.org/package=RColorBrewer). |
| *reshape2* | Hadley Wickham (2007). Reshaping Data with the reshape Package. Journal of Statistical Software, 21(12), 1-20. URL  http://www.jstatsoft.org/v21/i12/. |
| *rstatix* | Kassambara A (2023). rstatix: Pipe-Friendly Framework for Basic Statistical Tests. R package version 0.7.2, [https://CRAN.R-project.org/package=rstatix](https://cran.r-project.org/package=rstatix). |
| *tidyverse* | Wickham H, Averick M, Bryan J, Chang W, McGowan LD, François R, Grolemund G, Hayes A, Henry L, Hester J, Kuhn M, Pedersen TL, Miller E, Bache SM, Müller K, Ooms J, Robinson D, Seidel DP, Spinu V, Takahashi K, Vaughan D, Wilke C, Woo K, Yutani H (2019). “Welcome to the tidyverse.” Journal of Open Source Software, 4(43), 1686. doi:10.21105/joss.01686 <https://doi.org/10.21105/joss.01686>. |
| *tximeta* | Michael I. Love, Charlotte Soneson, Peter F. Hickey, Lisa K. Johnson, N. Tessa Pierce, Lori Shepherd, Martin Morgan, Rob Patro  Tximeta: Reference sequence checksums for provenance identification in RNA-seq PLOS Computational Biology 16(2): e1007664 |
| *tximport* | Charlotte Soneson, Michael I. Love, Mark D. Robinson (2015): Differential analyses for RNA-seq: transcript-level estimates improve  gene-level inferences. F1000Research |
| *UpSetR* | Gehlenborg N (2019). UpSetR: A More Scalable Alternative to Venn and Euler Diagrams for Visualizing Intersecting Sets. R package version 1.4.0, [https://CRAN.R-project.org/package=UpSetR](https://cran.r-project.org/package=UpSetR). |

# Strobe checklist

**STROBE Statement for MIS-ABC Sepsis**

Statement annotate from original source: <https://www.equator-network.org/reporting-guidelines/strobe/>

|  | Item No | Recommendation | Response |
| --- | --- | --- | --- |
| **Title and abstract** | 1 | (*a*) Indicate the study’s design with a commonly used term in the title or the abstract | We describe sub-study nature |
|  |  | (*b*) Provide in the abstract an informative and balanced summary of what was done and what was found | Format does not allow abstract |
| Introduction | | | |
| Background/rationale | 2 | Explain the scientific background and rationale for the investigation being reported | Provided in introduction |
| Objectives | 3 | State specific objectives, including any prespecified hypotheses | Provided in introduction, noting exploratory nature |
| Methods | | | |
| Study design | 4 | Present key elements of study design early in the paper | Explicitly included in introduction and methods |
| Setting | 5 | Describe the setting, locations, and relevant dates, including periods of recruitment, exposure, follow-up, and data collection | Included in methods, further information in main trial manuscript or protocol publications (referenced) |
| Participants | 6 | (*a*) *Cohort study*—Give the eligibility criteria, and the sources and methods of selection of participants. Describe methods of follow-up  *Case-control study*—Give the eligibility criteria, and the sources and methods of case ascertainment and control selection. Give the rationale for the choice of cases and controls  *Cross-sectional study*—Give the eligibility criteria, and the sources and methods of selection of participants | Included in methods, listed in supplementary materials |
|  |  | (*b*) *Cohort study*—For matched studies, give matching criteria and number of exposed and unexposed  *Case-control study*—For matched studies, give matching criteria and the number of controls per case |  |
| Variables | 7 | Clearly define all outcomes, exposures, predictors, potential confounders, and effect modifiers. Give diagnostic criteria, if applicable | Variables listed in methods |
| Data sources/ measurement | 8* | For each variable of interest, give sources of data and details of methods of assessment (measurement). Describe comparability of assessment methods if there is more than one group | Discussed in methods |
| Bias | 9 | Describe any efforts to address potential sources of bias | Main sources of bias and limitations discussed |
| Study size | 10 | Explain how the study size was arrived at | Exploratory study: no sample size calculation |
| Quantitative variables | 11 | Explain how quantitative variables were handled in the analyses. If applicable, describe which groupings were chosen and why | Summarised in methods, further detail in supplementary materials |
| Statistical methods | 12 | (*a*) Describe all statistical methods, including those used to control for confounding | Described in supplementary materials |
|  |  | (*b*) Describe any methods used to examine subgroups and interactions | Not applicable |
|  |  | (*c*) Explain how missing data were addressed | Described in supplementary materials |
|  |  | (*d*) *Cohort study*—If applicable, explain how loss to follow-up was addressed  *Case-control study*—If applicable, explain how matching of cases and controls was addressed  *Cross-sectional study*—If applicable, describe analytical methods taking account of sampling strategy | Visualised in supplementary materials – see missing data |
|  |  | (*e*) Describe any sensitivity analyses | Not applicable |
| Participants | 13* | (a) Report numbers of individuals at each stage of study—eg numbers potentially eligible, examined for eligibility, confirmed eligible, included in the study, completing follow-up, and analysed | Included in supplementary material visualisation Figure S1 |
|  |  | (b) Give reasons for non-participation at each stage | Included in supplementary material visualisation |
|  |  | (c) Consider use of a flow diagram | Alternative used |
| Descriptive data | 14* | (a) Give characteristics of study participants (eg demographic, clinical, social) and information on exposures and potential confounders | See table 1 |
|  |  | (b) Indicate number of participants with missing data for each variable of interest | Figure S1 |
|  |  | (c) *Cohort study*—Summarise follow-up time (eg, average and total amount) | Described fully in methods – does not vary |
| Outcome data | 15* | *Cohort study*—Report numbers of outcome events or summary measures over time | Provided in results |
|  |  | *Case-control study—*Report numbers in each exposure category, or summary measures of exposure | Not applicable |
|  |  | *Cross-sectional study—*Report numbers of outcome events or summary measures | Not applicable |
| Main results | 16 | (*a*) Give unadjusted estimates and, if applicable, confounder-adjusted estimates and their precision (eg, 95% confidence interval). Make clear which confounders were adjusted for and why they were included | Provided in results |
|  |  | (*b*) Report category boundaries when continuous variables were categorized | Not relevant to our variables |
|  |  | (*c*) If relevant, consider translating estimates of relative risk into absolute risk for a meaningful time period | Not relevant to the statistics in this manuscript |
| Other analyses | 17 | Report other analyses done—eg analyses of subgroups and interactions, and sensitivity analyses | Not applicable |
| Discussion |  |  |  |
| Key results | 18 | Summarise key results with reference to study objectives | Included in discussion |
| Limitations | 19 | Discuss limitations of the study, taking into account sources of potential bias or imprecision. Discuss both direction and magnitude of any potential bias | Included in limitations section of discussion |
| Interpretation | 20 | Give a cautious overall interpretation of results considering objectives, limitations, multiplicity of analyses, results from similar studies, and other relevant evidence | Included in discussion |
| Generalisability | 21 | Discuss the generalisability (external validity) of the study results | Included throughout discussion |
| Other information |  |  |  |
| Funding | 22 | Give the source of funding and the role of the funders for the present study and, if applicable, for the original study on which the present article is based | Included in statements section |

*Give information separately for cases and controls in case-control studies and, if applicable, for exposed and unexposed groups in cohort and cross-sectional studies.

**Note:** An Explanation and Elaboration article discusses each checklist item and gives methodological background and published examples of transparent reporting. The STROBE checklist is best used in conjunction with this article (freely available on the Web sites of PLoS Medicine at http://www.plosmedicine.org/, Annals of Internal Medicine at http://www.annals.org/, and Epidemiology at http://www.epidem.com/). Information on the STROBE Initiative is available at www.strobe-statement.org.

# Additional figures

## S1: Data completeness


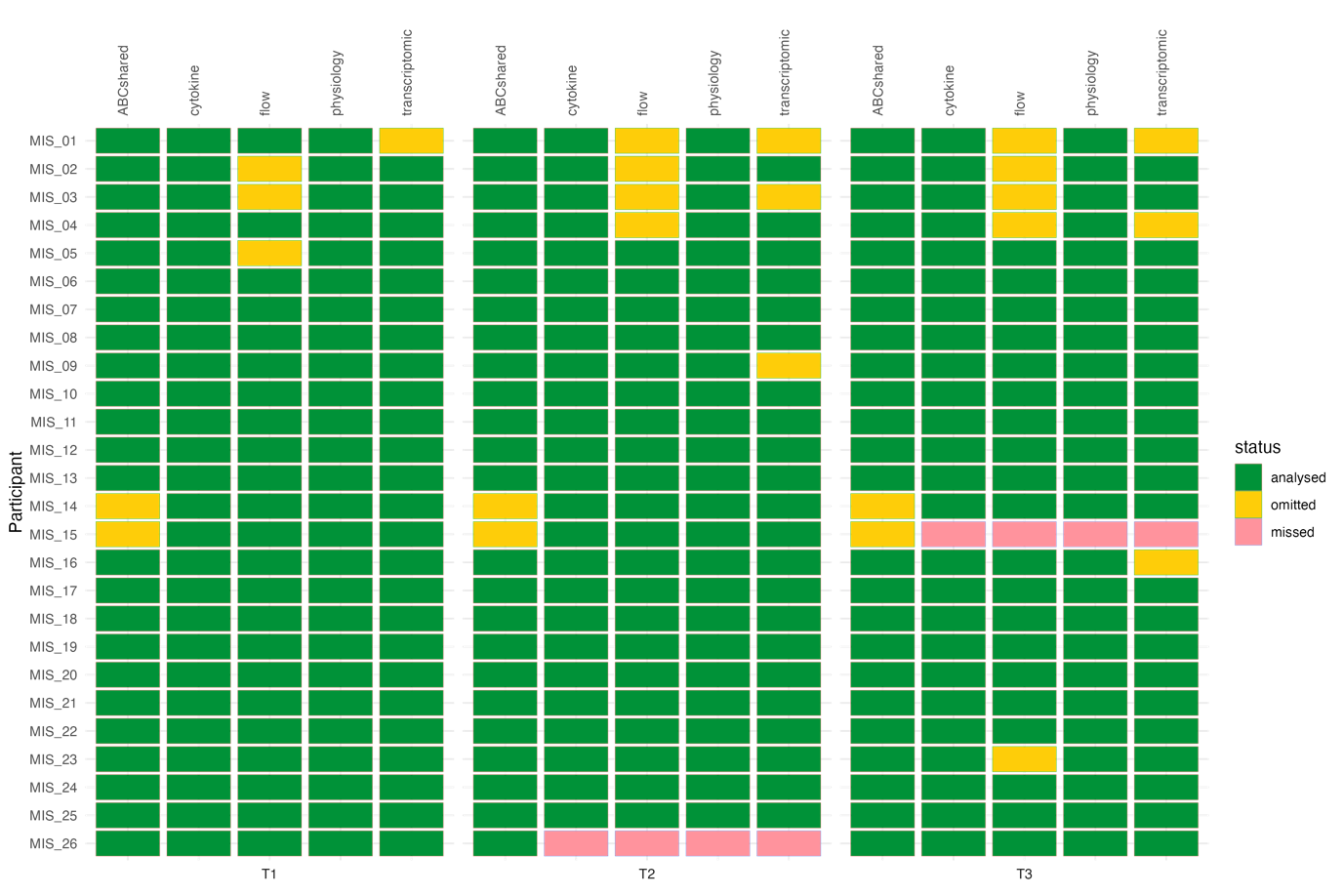


Figure S1: Illustration of data completeness

Each row describes a participant and columns describe a planned sample point, grouped into timepoints. The fill of the box describes completeness of the data: green describes datapoints which were collected and included in analysis; yellow describes datapoints collected but not included in the final analysis (in the case of ABCshared data, due to participants in the main trial not consenting for data sharing, for flow due to uninterpretable immunophenotyping results, and for transcriptomic due to insufficient genetic data in sample for sequencing). ABCshared refers to participant level data sharing between ABC Sepsis and MIS-ABC Sepsis. This visualisation demonstrates two samples missing: MIS-15 was unexpectedly discharged home prior to T3 sampling; and MIS-26 was unable to have T2 samples due to issues with site team availability out of hours.

## S2: Differential expression analysis

Tabulated results of genes identified as having differential expression at stated timepoints, with thresholds of p_adj_ < 0.05 and absolute Log_2_FoldChange > 1.5.

Comparison between groups at baseline:

| **external_gene_name** | **log2FoldChange** | **padj** |
| --- | --- | --- |
| BCAR1 | 3.060345 | 0.046341 |
| CELSR1 | 1.946026 | 0.046341 |
| SIGLEC1 | -3.95666 | 0.010319 |
| EPB41L4B | -6.71569 | 0.03493 |
| DERL3 | 2.072178 | 0.046801 |
| CCNE1 | 1.691027 | 0.046801 |
| NCAPH | 1.658648 | 0.017018 |
| B4GALNT3 | 2.248692 | 0.046249 |
| ROBO4 | 2.285595 | 0.049458 |
| IGLV2-23 | 3.097588 | 0.010374 |
| IGLC1 | 2.8774 | 0.046249 |
| IGLC2 | 3.120745 | 0.021319 |
| TCL1B | 4.083004 | 0.006329 |
| CPSF1P1 | 22.746 | 0.004201 |
| TBC1D3K | 18.1347 | 0.00023 |
| RN7SL1 | 1.89426 | 0.049458 |
| IGLV2-8 | 3.075777 | 0.046341 |
| U2 | 20.96147 | 0.001133 |

5% HAS group T2 versus T1 had no relevant genes. 5% HAS T3 versus T1:

| **external_gene_name** | **log2FoldChange** | **padj** |
| --- | --- | --- |
| TEAD3 | -2.67759 | 0.04971 |
| ATP2C2 | -3.38003 | 0.00028 |
| DUSP13 | -2.00133 | 0.04971 |
| OLFM4 | -3.42698 | 0.042327 |
| AREG | -2.75941 | 0.038728 |
| MMP8 | -3.61287 | 0.0004 |
| G0S2 | -2.43137 | 0.008981 |
| TRIB1 | -1.70035 | 0.0004 |
| HBA1 | -4.74902 | 0.04971 |
| HBB | -4.44119 | 0.002336 |
| MARCKS | -1.53617 | 0.005424 |

Crystalloid T2 versus T1:

| **external_gene_name** | **log2FoldChange** | **padj** |
| --- | --- | --- |
| FKBP4 | -2.09811 | 0.009396 |
| PITPNM3 | 1.915305 | 0.013263 |
| HSPB1 | -2.56751 | 0.001862 |
| OTOF | 3.573156 | 0.013718 |
| HSPH1 | -1.97088 | 0.02415 |
| VSIG4 | 2.605934 | 0.02415 |
| HSPA1A | -2.26407 | 0.001847 |
| TBC1D3K | 18.92026 | 0.001494 |
| U2 | 19.95592 | 0.013718 |

Crystalloid T3 versus T1:

| **external_gene_name** | **log2FoldChange** | **padj** |
| --- | --- | --- |
| FKBP4 | -2.18682 | 0.000590 |
| ATP2C2 | -3.36705 | 0.000685 |
| SLC1A3 | -3.31114 | 0.008760 |
| CEACAM1 | -2.79068 | 0.016696 |
| HSP90AA1 | -2.18719 | 0.000200 |
| HSP90AB1 | -1.58829 | 0.005394 |
| HSPB1 | -2.51353 | 0.000539 |
| EPAS1 | -1.94953 | 0.007077 |
| HSPH1 | -2.10071 | 0.001918 |
| CPNE5 | -1.75699 | 0.021829 |
| UGCG | -2.21686 | 0.007635 |
| TRIB1 | -1.97926 | 0.000074 |
| TNFRSF10D | -1.55852 | 0.045004 |
| SOCS3 | -2.1219 | 0.030578 |
| HSPA1A | -2.42675 | 0.000074 |
| *NA* | -2.48104 | 0.011876 |
| LINC01093 | -2.91746 | 0.039755 |
| CLEC5A | -2.32352 | 0.004725 |

## S3: Blood tests

|  | **5% Human Albumin Solution** | **Balanced Crystalloid** | **Overall** |
| --- | --- | --- | --- |
| **Baseline laboratory results** | | | |
| Haemoglobin (g/L) | 136 (119, 146) | 130 (112, 144) | 131 (116, 144) |
| Leucocytes (×10⁹/L) | 11.5 (7.8, 14.4) | 16.9 (9.1, 18.8) | 14.0 (9.0, 18.1) |
| Neutrophils (×10⁹/L) | 8.66 (5.40, 12.80) | 14.90 (8.20, 16.02) | 11.05 (7.20, 15.67) |
| Platelets (×10⁹/L) | 223 (137, 271) | 271 (141, 366) | 232 (140, 312) |
| Urea (mmol/L) | 6.5 (4.2, 11.1) | 6.1 (4.7, 10.7) | 6.3 (4.5, 10.9) |
| Creatinine (μmol/L) | 93 (75, 106) | 78 (75, 116) | 90 (75, 111) |
| Albumin (g/L) | 32 (28, 35) | 33 (28, 36) | 32 (28, 36) |
| ALT (U/L) | 24 (20, 34) | 15 (11, 16) | 20 (13, 34) |
| CRP (mg/L) | 46 (17, 133) | 52 (14, 127) | 49 (17, 130) |
| **Collection times** (h) | | | |
| T1 | 1.59 (0.73, 3.00) | 1.41 (1.08, 1.96) | 1.54 (0.98, 2.53) |
| T2 | 11.25 (10.27, 12.82) | 11.43 (9.98, 13.92) | 11.28 (10.27, 12.82) |
| T3 | 25.95 (23.97, 26.90) | 24.68 (24.13, 25.77) | 25.29 (24.05, 26.57) |

Figure S3: Blood test information. Baseline laboratory results results were collected by the main trial and subsequently shared with this substudy, relying on written participant consent to do so. This was not present for one participant in each group, meaning laboratory data are not able to be included in this table, resulting in an N=13 (rather than 14) for 5% Human Albumin Solution group and N=11 (rather than 12) for Balanced Crystalloid. Collection times represent the time from presentation to the Emergency Department to sampling, measured in hours (where 1.50 hours = 90 minutes). Both laboratory results and collection times are presented as median (Q1, Q3).

1. Personal correspondence [↑](#footnote-ref-1)
2. Maecker HT, McCoy JP, Nussenblatt R. Standardizing immunophenotyping for the Human Immunology Project. Nat Rev Immunol. 2012 Feb 17;12(3):191-200. doi: 10.1038/nri3158. Erratum in: Nat Rev Immunol. 2012 Jun;12(6):471. PMID: 22343568; PMCID: PMC3409649. [↑](#footnote-ref-2)
3. Parks DR, Roederer M, Moore WA. A new "Logicle" display method avoids deceptive effects of logarithmic scaling for low signals and compensated data. Cytometry A. 2006 Jun;69(6):541-51. doi: 10.1002/cyto.a.20258. PMID: 16604519. [↑](#footnote-ref-3)
4. Precision Count Beads™ Protocol and Applications, BioLegend. <https://www.biolegend.com/protocols/precision-count-beads-protocol-and-applications/4248/> [↑](#footnote-ref-4)
5. Shuzhao Li “BTM” <https://github.com/shuzhao-li/BTM?tab=readme-ov-file> [↑](#footnote-ref-5)
